# FishKP-YOLOv11: An Automatic Estimation Model for Fish Size and Mass in Complex Underwater Environments

**DOI:** 10.3390/ani15192862

**Published:** 2025-09-30

**Authors:** Jinfeng Wang, Zhipeng Cheng, Mingrun Lin, Renyou Yang, Qiong Huang

**Affiliations:** 1 College of Mathematics and Informatics, South China Agricultural University, Guangzhou 510642, China; wangjinfeng@scau.edu.cn (J.W.); 1121659938@stu.scau.edu.cn (M.L.); 2 Southern Marine Science and Engineering Guangdong Laboratory (Zhanjiang), Zhanjiang 524000, China; youngrenyou@zjblab.com

**Keywords:** computer vision, key point detection, stereo vision, fish size estimation, fish mass estimation

## Abstract

The size and mass of fish are crucial parameters in aquaculture management. However, existing research primarily focuses on conducting fish size and mass estimation under ideal conditions, which limits its application in actual aquaculture scenarios with complex water quality and fluctuating lighting. A non-contact size and mass measurement framework is proposed for complex underwater environments, which integrates the improved FishKP-YOLOv11 module based on YOLOv11, stereo vision technology, and a Random Forest model. This framework fuses the detected 2D key points with binocular stereo technology to reconstruct the 3D key point coordinates. Fish size is computed based on these 3D key points, and a Random Forest model establishes a mapping relationship between size and mass. For validating the performance of the framework, a self-constructed grass carp dataset for key point detection is established. The experimental results indicate that the mean average precision (mAP) of FishKP-YOLOv11 surpasses that of diverse versions of YOLOv5–YOLOv12. The mean absolute errors (MAEs) for length and width estimations are 0.35 cm and 0.10 cm, respectively. The MAE for mass estimations is 2.7 g. Therefore, the proposed framework is well suited for application in actual breeding environments.

## Simple Summary

Fish size and mass are crucial parameters for scientific feeding and harvesting in aquaculture. This paper proposes a non-contact framework for estimating fish size and mass. The framework addresses inaccurate size and mass estimation in actual aquaculture scenarios, which are often caused by complex water quality, low illumination, and high stocking densities. The proposed framework achieves accurate size and mass estimation in complex underwater environments. The experimental results demonstrate that the framework achieves high estimation accuracy and strong practical applicability. The framework can be deployed in an actual aquaculture scenario to reduce labor requirements, decrease fish stress, and improve yield and income for producers.

## 1. Introduction

Aquaculture is a major producer of aquatic products and contributes significantly to global food and nutrition security. It is expected to expand further owing to the increasing global population and rising affluence [1]. Information on fish size and mass is crucial in aquaculture. It supports precise feeding, optimization of stocking density, and growth prediction [2,3,4]. Traditional methods rely on direct human contact to measure fish size and mass. Those methods are time-consuming and can induce stress responses in fish. Stress can reduce growth rate and compromise health [5,6]. Therefore, non-contact methods for measuring fish size and mass are urgently required. Such methods can replace contact-based approaches and reduce human intervention, thereby improving aquaculture efficiency.

Machine learning and deep learning have been widely applied across numerous domains and have been used to address many core problems [7,8,9,10]. In aquaculture, these techniques have been employed to extract fish body features and to enable non-contact size and mass monitoring [11,12,13,14]. From a computer-vision perspective, non-contact size and mass estimation methods fall into two categories: monocular-vision approaches [15,16] and stereo-vision approaches [17,18].

Monocular cameras offer a simple structure and low cost. Methods based on monocular cameras typically constrain the swimming direction of the fish or place a reference object of a known real size beside the fish to map pixel measurements to physical dimensions [19,20,21]. Length measurements of European sea bass were obtained using ArUco fiducial markers attached to polypropylene plates [22]. The Principal Component Analysis–Calibration Factor (PCA–CF) method was applied to extract image features, and the Backpropagation Neural Network (BPNN) was employed to estimate fish mass [11]. Other studies extracted fish length, mass, and color from images and the length–mass relationship through the application of mathematical models [23]. Although monocular methods provide beneficial results, they generally require fixing fish on a specific plane or using reference objects. Such requirements restrict these methods to single-fish measurements and limit their applicability in real-world environments. To address this limitation, stereo-vision techniques have been adopted to estimate the size and mass of freely swimming fish underwater.

Compared to two-dimensional vision, stereoscopic vision can wholly and accurately restore the three-dimensional spatial information of objects. This capability enables high-dimensional feature extraction [24,25,26]. In aquaculture, binocular stereoscopic vision captures fish images via left and right cameras. It then employs stereo matching algorithms to calculate the depth of the fish body. This process achieves the acquisition of three-dimensional fish body data without the need for setting reference objects or fixing the position of the fish. Consequently, it realizes a precise estimation of fish size and mass. This technology facilitates non-contact dynamic measurement of size and mass in free-swimming fish within modern intelligent aquaculture systems [27,28,29].

The CamTrawl system processes fish images via background subtraction and combines binocular stereo vision technology with the triangulation model to estimate fish length [30]. Local threshold segmentation and geometric model fitting algorithms were employed to process fish body images. Combined with a deformable ventral contour model and binocular stereo vision technology, tuna length was estimated [31]. The underwater non-contact method for estimating the mass of free-swimming fish, developed based on the LabVIEW platform, estimates fish mass by integrating binocular stereo vision technology with the linear model of fish area [32]. These methods can quickly and accurately extract fish features. However, in complex underwater environments, issues such as diverse fish postures and light refraction arise. Sole reliance on mathematical fitting methods may reduce feature extraction accuracy, thereby increasing errors in estimating fish size and mass.

To further improve the accuracy of fish feature extraction, deep learning has been integrated with binocular vision technology to estimate fish size and mass. The key points of fish bodies were extracted by utilizing the key point detection network key points R-CNN. Three-dimensional information of these key points was then obtained via 3D reconstruction of the fish body. Finally, fish body length was calculated using the three-dimensional Euclidean distance [33]. Deep stereo matching based on Neural Radiance Fields (NeRFs) has been applied for the accurate depth estimation of fish surfaces. When coupled with instance segmentation, this approach yields edge key points for bass, which are used to compute perimeters and estimate mass [34]. An SE-D-KP-RCNN network has been proposed for fish key point detection, with integration with CREStereo deep stereo matching, enabling the fitting of spatial planar curves to derive fish dimensions [35]. Instance-segmentation methods have been used to extract fish contours; Principal Component Analysis (PCA) then corrects the orientation to obtain key points, and point-cloud processing combined with XGBoost is applied to estimate fish mass [36]. Multi-task networks capable of concurrently executing key point detection, segmentation, and stereo matching have been developed to extract morphological features. Subsequently, a segmented fitting strategy was employed for body length measurement [37]. Although these deep learning methods have improved the accuracy of fish feature extraction, factors such as fish body overlap, occlusion, body curvature, and adhesion affect feature extraction in dense aquaculture environments [38]. Moreover, in practical aquaculture scenarios, the data often contains a great deal of uncertainty [39]. Rapid changes in illumination and increased water turbidity—frequently caused by suspended white flocculent matter—degrade the quality of captured fish images and hinder reliable feature extraction [40,41]. These problems affect the accuracy and stability of feature extraction, further influencing the estimation accuracy of fish body size and mass.

Previous studies were predominantly conducted under ideal conditions characterized by low stocking density, stable lighting, and clear water. Consequently, these methods are ill equipped to handle the complex challenges encountered in real-world aquaculture settings, such as high stocking densities, abrupt lighting fluctuations, and turbid water. Some approaches have estimated fish length from surface images; however, surface-based methods cannot meet the requirements for real-time underwater measurement of fish length and mass in deep-sea aquaculture. To overcome these limitations, this paper proposes FishKP-YOLOv11, a novel framework for estimating fish length and mass. Trained on datasets spanning diverse water conditions, illumination levels, and stocking densities, the framework enables automatic, accurate, and non-contact estimation of size and mass for free-swimming fish in complex, dynamic aquaculture environments. The main contributions of this paper are listed as follows:

(1) A dataset is collected and constructed with a binocular camera for shooting grass carp with different conditions, containing 2674 images with corresponding annotation information. The self-built dataset makes up for the problem of a lack of data.

(2) A feature extraction model, HGCBlock (composed of HCEBlock and GDCBlock), was designed to capture discriminative fish features under challenging conditions. A Feature Pyramid Network (FPN) was incorporated to catch the fish’s edges and textures while reducing channel-wise redundancy and computations. The Head network was redesigned to improve both localization accuracy and classification performance.

(3) A framework based on the improved YOLOv11 is proposed for estimating fish size and mass, called FishKP-YOLOv11. The detected key points are fused with binocular stereo technology to recover the three-dimensional coordinates. A Random Forest regression model is introduced to map the computed size features to fish mass. The proposed framework realized accurate and non-contact estimation of fish size and mass in complex aquaculture environments.

## 2. Materials and Methods

### 2.1. Data Acquisition

**Figure 1:**
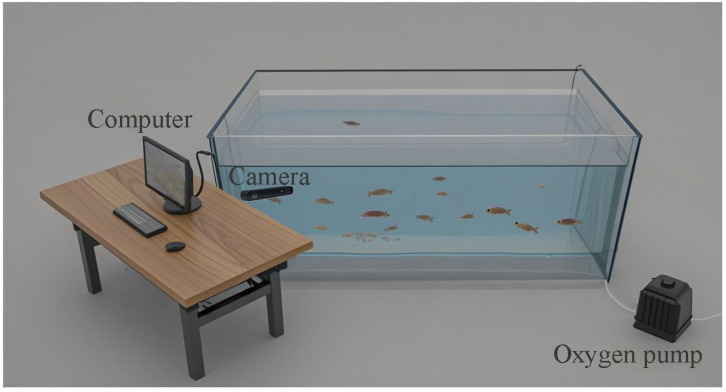
Experimental scenario of this work.

To collect underwater grass carp (*Ctenopharyngodon idella*) image data in a real aquaculture environment, this study designed an aquaculture system that can simulate the underwater ecological environment in nature. The actual aquaculture scene is shown in Figure 1. The main devices for data collection include a water tank with 2.5 × 1.5 × 0.9 m dimensions, an oxygenator, a ZED 2i stereo camera with a baseline of 120 mm and a focal length of 2.12 mm, an electronic scale with an accuracy of 0.1 g, a vernier caliper with an accuracy of 0.01 mm, and a black and white grid calibration board with 0.4 × 0.3 m.

**Figure 2:**
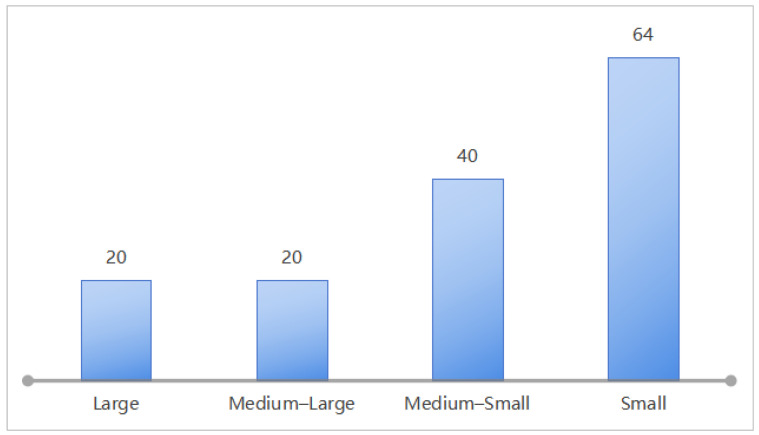
Quantities of grass carp with varying sizes.

In this study, 144 grass carp were obtained from a licensed aquatic farm. The fish were acclimated in the aquaculture system for seven days and were fed a formulated diet daily to ensure normal growth. During the experiment, a ZED 2i binocular camera was placed in a transparent waterproof case and then installed in the water tank to capture videos of freely swimming grass carp. Recorded video data were transmitted to a computer via a data transmission cable and stored for subsequent analysis. Video resolution was 1920 × 1080 pixels at 30 frames per second. After the video data recording was completed, fish were anesthetized with 100 mg/L tricaine methanesulfonate (MS-222) immediately after removal from the tank to minimize handling stress. To ensure measurement accuracy, the length and width of each fish (excluding the tail) were measured three times using a vernier caliper with 0.01 mm precision. The body mass was then measured three times using an electronic scale with a precision of 0.1 g. The average of the repeated measurements was taken as the actual value. Measurements showed a maximum body length (excluding tail) of approximately 0.21 m and a minimum of approximately 0.06 m. All fish were categorized into four classes, which are shown in Figure 2. Following the experiment, the fish were kept in the institution’s aquaculture system for long-term breeding and were reserved for subsequent non-invasive research.

**Figure 3:**
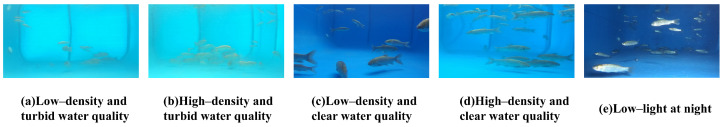
Experimental data obtained from diverse environments and diverse stocking densities. Due to the natural variability in outdoor light and the automatic adjustment of the camera’s white balance, the colors of pictures taken at different times are inconsistent.

To reflect realistic aquaculture conditions, recordings relied exclusively on natural light during daytime without supplementary lighting. No water filters were applied to preserve the clearest water quality, and only partial water changes were performed to maintain fish health. Under fully dark conditions at night, an LED lamp inside the tank provided the sole illumination for low-light video capture. Consequently, video data were collected across a range of lighting intensities, stocking densities, and water turbidities. As shown in Figure 3, the dataset includes low-density with clear water, high-density with clear water, low-density with turbid water, high-density with turbid water, and low-light conditions. Across three experimental cycles, eighty videos were recorded, comprising single fish, groups of similarly sized fish, and mixed-sized groups. This data collection strategy ensures dataset diversity and realism, and provides a robust foundation for subsequent analyses.

### 2.2. Dataset Construction

Within the key point detection dataset, key points and bounding boxes were annotated only for fish oriented approximately parallel to the imaging plane. Fish with occluded key points or pronounced body curvature were excluded from annotation. In total, 2674 valid images—covering diverse lighting conditions, water qualities, stocking densities, and fish sizes—were selected from multiple videos for labeling. The specific image counts for each condition are presented in Table 1. To reduce subjective bias, three annotators received uniform training and independently labeled the 2674 frames using LabelMe for both bounding boxes and key points. Following the labeling process, the annotators completed three rounds of review to remove fish that were not parallel to the imaging plane and to correct missed or mislabeled key points. The dataset was then split into training, validation, and test subsets at a ratio of 7:1:2. The dataset is available at https://github.com/pigcat88/FishKP-YOLOv11.git (accessed on 28 August 2025). Additionally, separate tests were carried out using single-fish images and groups of fish with similar size selected from outside the key point detection dataset to validate the accuracy of size and mass predictions and to verify the reliability of the results.

### 2.3. Experimental Setup

The experimental environment for the key point detection model is presented in Table 2. During training, the SGD optimizer was employed with an initial learning rate of 0.01, a weight decay of 0.01, and a total of 150 training epochs. To prevent overfitting, training was halted 50 epochs in advance if there was no improvement in the validation metric.

### 2.4. Overall Structure of the Proposed Method

**Figure 4:**
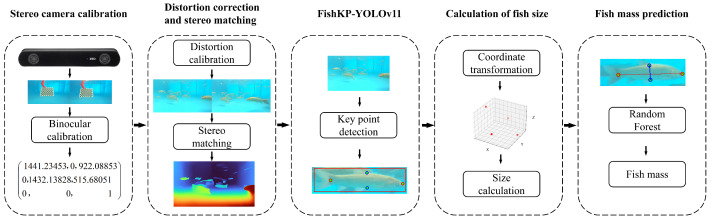
Overall process of the proposed method.

In practical aquaculture scenarios, variable water quality and illumination cause a reduction in image clarity. Blurred images hinder the extraction of fish body features and decrease the accuracy of size and mass estimation. Under high-density stocking conditions, freely swimming fish frequently change posture. Overlap, occlusion, body bending, and adhesion further complicate feature extraction. To address these challenges, this paper proposes a method that automatically and accurately estimates the size and mass of freely swimming grass carp under complex water quality and lighting conditions. The overall workflow is illustrated in Figure 4 and summarized below:

(1) Construct an experimental data-acquisition system. A ZED 2i binocular camera, mounted in the system, recorded videos of freely swimming fish. Images representing different water qualities, lighting conditions, and stocking densities were randomly sampled in proportion from the recorded videos. Fish body key points and bounding boxes were annotated on the sampled images to construct a fish key point detection dataset.

(2) Train the FishKP-YOLOv11 model based on the fish key point detection dataset. The trained model detects fish bodies that are approximately parallel to the camera plane and that present complete, non-occluded key points.

(3) Calculate the size of the fish. Following the transformation of two-dimensional pixel coordinates of key points into three-dimensional spatial coordinates, the body measurements of the fish can be calculated based on the three-dimensional Euclidean distances between these points within the spatial coordinate system.

(4) Build a fish mass estimation model. The size of the fish serves as input to the Random Forest algorithm for predicting the fish body mass.

### 2.5. Key Point Detection Model

#### 2.5.1. Structure of FishKP-YOLOv11

YOLOv11 [42], the latest release in the Ultralytics YOLO series, provides high precision, high speed, and efficient object and key point detection capabilities. Its network architecture comprises three components: the backbone, Neck, and Head. The model adopts channel separation principles, fuses channel and spatial information, and employs an improved spatial-pyramid-pooling module; these design choices preserve a lightweight footprint while strengthening feature extraction capacity. Additionally, YOLOv11 employs refined architectural configurations and optimized training pipelines to achieve faster processing speeds while maintaining a balance between accuracy and performance. Unlike other YOLO versions, YOLOv11 provides native support for both object detection and key point detection and is distributed as a complete framework by Ultralytics. The optimized architecture and efficient processing make the model suitable for deployment on edge devices, cloud platforms, and systems equipped with NVIDIA GPUs. This deployment flexibility enhances the practical applicability of YOLOv11 in real-world aquaculture environments, making it particularly well suited for fish key point detection compared to other YOLO variants.

**Figure 5:**
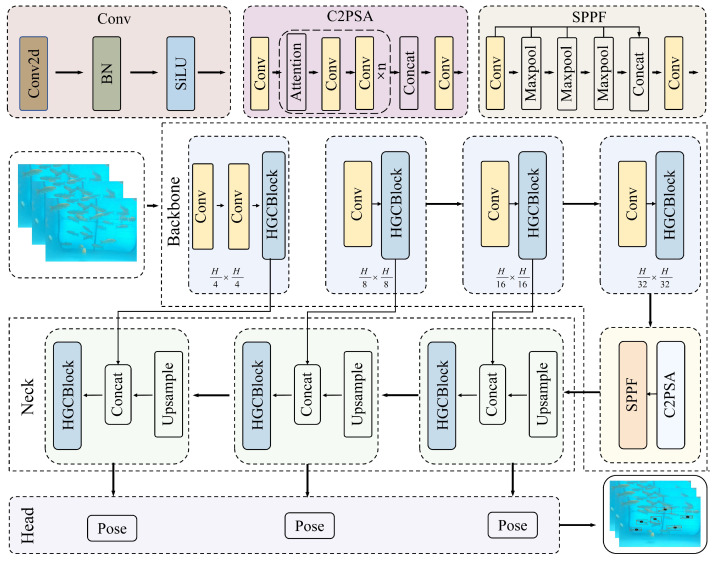
Structure of FishKP-YOLOv11.

However, the model was not developed specifically for underwater scenes. Underwater factors, such as light refraction and increased turbidity, can substantially degrade detection performance. To improve detection accuracy in complex underwater environments while preserving a lightweight model for practical deployment, the most lightweight YOLOv11n was selected as the base. Building on this base and accounting for underwater imaging characteristics and fish morphology, the FishKP-YOLOv11 model was developed. The model’s overall framework is shown in Figure 5. The Hybrid Gated Convolutional Block (HGCBlock) functions as the primary feature extraction unit. HGCBlock comprises two submodules, the Hybrid Convolutional Enhancement Block(HCEBlock) and the Gated Depthwise Convolutional Block(GDCBlock), which enable more thorough extraction of fish-specific features. Feature fusion in the Neck was improved by integrating the Feature Pyramid Network (FPN) [43], which reduces redundancy from repeated fusion and helps improve detection accuracy. Deep convolutional layers [44] were added to deepen the Head network; this change increases the detection Head’s resolution capability while controlling the model’s parameter count.

Images captured at 1920 × 1080 pixels are resized to 640 × 640 pixels and then fed into the FishKP-YOLOv11 backbone to reduce computational load and improve detection efficiency. The Conv and HGCBlock modules in the backbone extract multi-level features. The C2PSA module, which incorporates an attention mechanism and convolutional layers, further enhances feature extraction and captures deeper semantic information. The SPPF spatial-pyramid-pooling module integrates multi-scale information and strengthens the model’s ability to detect fish of varying sizes. In the Neck, the Feature Pyramid Network (FPN) fuses features across scales and improves overall feature fusion effectiveness. Finally, the multi-layer deep convolutional Head predicts bounding boxes, class labels, and key points for each detected fish.

#### 2.5.2. Structure of HGCBlock

**Figure 6:**
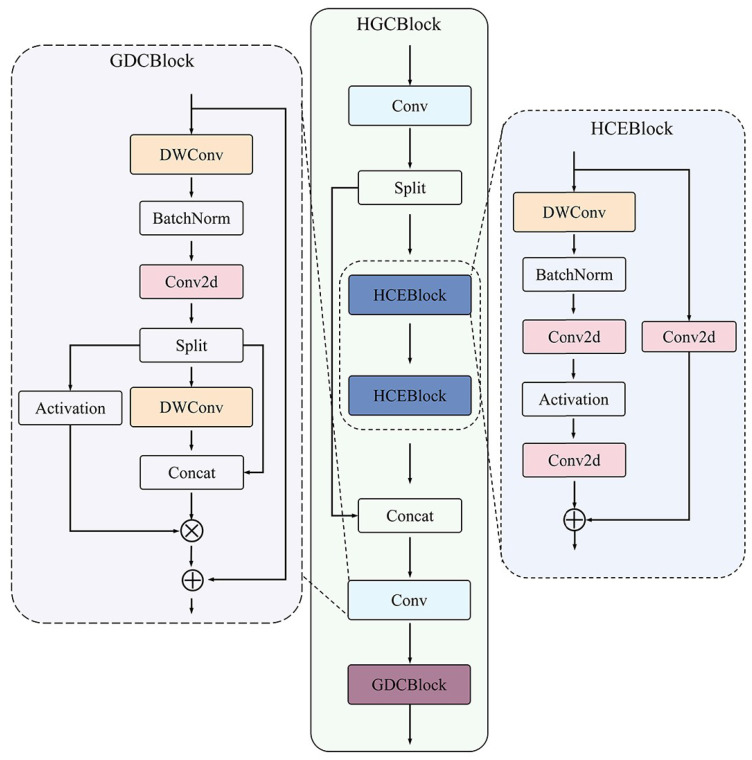
Structure of HGCBlock.

To extract effective fish feature information from complex aquaculture environment conditions, this paper designs a feature extraction module—HGCBlock—shown in Figure 6, which is the core component of FishKP-YOLOv11. Drawing on the idea of CSPNet [45], the design of the HGCBlock module adheres to the gradient flow splitting and merging strategy to enhance the network’s learning ability and reduce computational load. The HGCBlock module can be decoupled into two submodules: HCEBlock and GDCBlock. The features first enter a Conv module for preliminary convolution processing, and then are split into two parts through channel separation. One part is enhanced through the HCEBlock module, and the resulting features are fused with the other part of the original features to obtain a more comprehensive feature representation. The new features are integrated through a Conv module and then enter the GDCBlock module for gated weight feature adjustment to enhance the expressiveness of the features, ultimately outputting a more refined feature map.

Actual aquaculture scenarios demand lightweight, fast, and accurate models. The HCEBlock module targets improved feature extraction efficiency and accuracy. The module adopts a dual-branch residual architecture. The main branch begins with a deep convolution layer and then applies BatchNorm, a standard convolution, and an activation function. This branch extracts deep features, increases feature dimensionality, and enhances nonlinear representation. The residual branch applies a standard convolution to capture shallow features. Outputs from both branches are fused to yield a feature map with a more comprehensive representation. HCEBlock employs this dual-branch hybrid convolution strategy to preserve fine-grained details while improving semantic understanding. Deep convolutional operations further increase feature extraction efficiency. These design choices improve detection accuracy and inference speed in complex aquaculture environments.

Overlapping, occlusion, body bending, and adhesion produce background fish whose features closely resemble those of target fish that are oriented parallel to the camera plane and have complete key points. Thus, the model must distinguish subtle differences between these two types of fish bodies. To equip the model with this capability, a gated dynamic weighting mechanism and a residual fusion strategy were introduced during the design of the GDCBlock module. The gated unit dynamically adjusts feature weights to emphasize key features of the target fish body. When combined with the residual fusion strategy, this approach retains more detailed information. This enhances the model’s ability to recognize subtle differences in fish bodies and ensures accurate detection in complex environments. The GDCBlock module first extracts feature information via deep convolution. It then normalizes these features and performs ordinary convolution to adjust the channel dimension and fuse feature information, leading to the decoupling of features into three branches. The first branch undergoes activation processing to serve as a dynamic weight signal. To ensure the feature map contains both fine-grained and coarse-grained features—thereby enhancing feature discriminability—the second branch extracts spatial features via deep convolution. It is then concatenated with the third branch, which contains the original features. The resulting features are weighted and fused using gated weights. They are then connected to the original features in a residual manner, alleviating the gradient vanishing problem caused by multiple transformations in the main branch. Dynamically weighting features via the gated unit effectively improves the model’s recognition accuracy for target fish bodies in complex aquaculture environments.

#### 2.5.3. Structure of FPN

**Figure 7:**
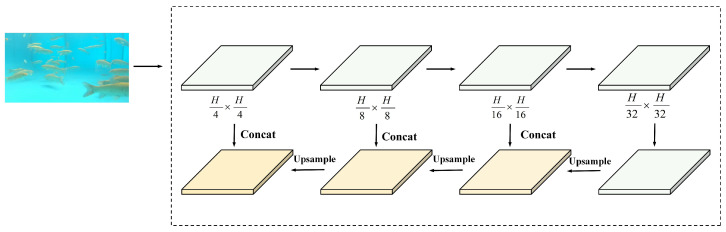
Structure of FPN.

The Feature Pyramid Network (FPN) in the Neck serves as the core module for addressing multi-scale object detection. By fusing features from deep and shallow layers, the FPN enables the model to detect both small and large targets. Because near objects appear larger and distant objects appear smaller, fish exhibit substantial scale variation at different distances; therefore, feature extraction at multiple scales is required. As features of background fish and target fish are highly similar, the repeated interaction of multi-directional and multi-scale features enhances multi-scale feature representation. However, it also causes information overlap in the channel dimension and redundant feature calculations, leading to false detections. To mitigate these issues, this study adopts the FPN-based fusion strategy shown in Figure 7. The FPN progressively fuses feature maps across levels to construct multi-scale representations and reduce false detections caused by repeated fusion, thereby enhancing the model’s ability to capture the edges and textures of fish bodies. At the same time, the incorporation of shallow features preserves fine-grained details and helps recover performance lost to image blurring.

#### 2.5.4. Structure of the Detection Head

**Figure 8:**
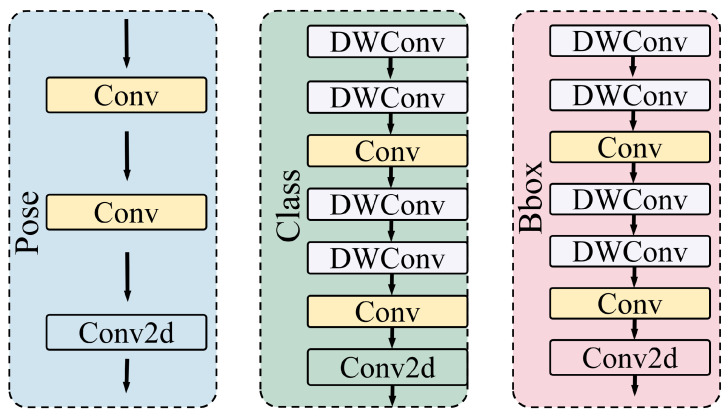
Structure of Head.

The Head network is responsible for processing multi-scale features and generating the final detection results. In complex aquaculture scenarios, detecting fish body key points depends on accurate classification of the target fish from the background and precise regression of the bounding boxes. To address this, this study introduces a double-layer deep convolution structure into both the classification and bounding box regression branches of the Head network, thereby increasing network depth. The structure of the Head is shown in Figure 8. This approach enhances the model’s performance in both classification and bounding box regression while simultaneously reducing the number of model parameters. This ensures that the model remains lightweight, making it suitable for deployment in real-world aquaculture scenarios.

### 2.6. Stereo Camera Calibration

**Figure 9:**
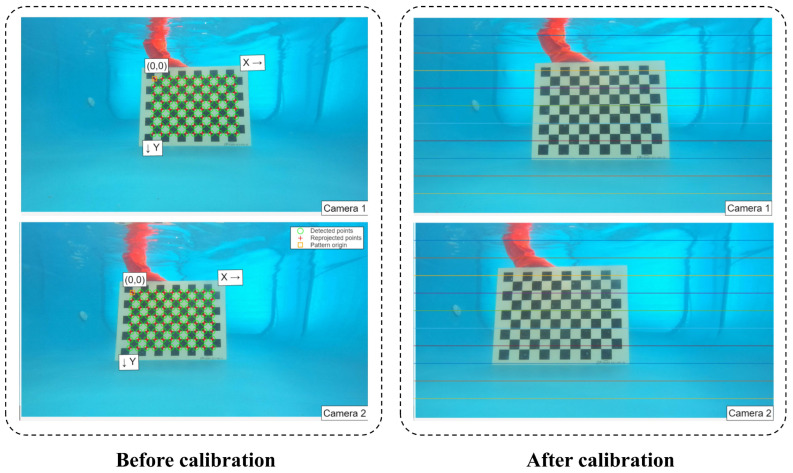
Comparison before and after the calibration of the (**left**,**right**) images.

This paper utilizes the stereo camera calibration module provided by MATLAB R2024a to calibrate the ZED 2i binocular camera. During the calibration process, checkerboard images captured at 1 m, 1.5 m, and 2 m underwater are input into MATLAB R2024a to calculate the reprojection error for each image pair. By manually removing image pairs with larger errors, the calibration accuracy is ensured. Ultimately, 30 pairs of images with an average error of 0.1 pixels are obtained. The comparison of the left and right images before and after calibration is shown in Figure 9. The corresponding pixel points align along the same horizontal line, demonstrating the effectiveness of the calibration. Distortion is effectively corrected, providing a reliable foundation for subsequent stereo matching and the calculation of three-dimensional spatial coordinates.

### 2.7. Size and Mass Estimation

#### 2.7.1. Key Point Setting

**Figure 10:**
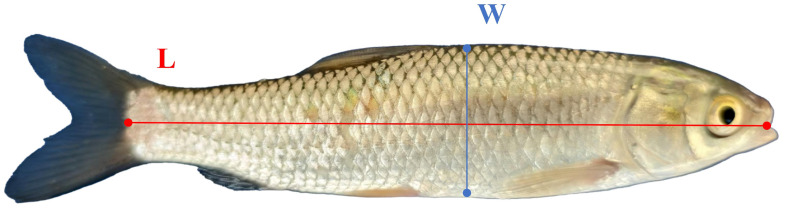
Example of key points for grass carp. L and W represent the length and width of the fish body, respectively. Considering that the bending and swinging of the tail fin will affect the estimation of its length, and its contribution to mass is relatively small; the tail fin is not taken into account when setting the key points.

The length and width of fish are strongly correlated with their mass, a relationship that numerous studies have confirmed through experiments [46,47,48]. Therefore, in this study, four key points were identified: the Head point, the tail point, the upper width point, and the lower width point. The positions of these key points are illustrated in Figure 10. The line connecting the Head point and the tail point represents the length of the fish body, while the line connecting the upper width point and the lower width point represents the width of the fish body.

#### 2.7.2. Coordinate Transformation of Key Points

**Figure 11:**
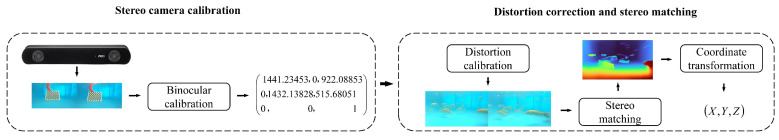
Process of generating three-dimensional spatial coordinates.

The key point information of the fish body detected by the FishKP-YOLOv11 key point detection model is two-dimensional, representing the coordinates of the key points on the 2D image. Relying solely on this 2D information does not provide the distance of the fish body from the camera plane. Therefore, it is necessary to obtain the depth information of the key points through a stereo matching algorithm, combined with the camera’s internal and external parameters, to construct three-dimensional spatial positions. The process for generating three-dimensional spatial coordinates is illustrated in Figure 11 and summarized below:

(1) Capture images of the underwater checkerboard calibration board and apply Zhang’s calibration technique [49] to obtain the camera’s internal and external parameters;

(2) Use these internal and external parameters to correct the distortion in the recorded underwater fish body images;

(3) Utilize the End-to-End Neural disparity estimation function NEURAL provided by the ZED SDK to obtain the depth information for the fish body key points;

(4) Combine the depth information, 2D coordinates, and camera internal and external parameters to convert the 2D coordinates of the fish body key points into 3D spatial coordinates. The conversion formula is presented in Equation (1):

(1) $$z\left[\begin{array}{c} u\\ v\\ 1 \end{array}\right]=\left[\begin{array}{cccc} f_{x} & 0 & u_{0} & 0\\ 0 & f_{y} & v_{0} & 0\\ 0 & 0 & 1 & 0 \end{array}\right]\left[\begin{array}{cc} R & T\\ \vec{0} & 1 \end{array}\right]\left[\begin{array}{c} X\\ Y\\ Z\\ 1 \end{array}\right].$$

Specifically, *z* represents the distance of the key point from the camera plane. *u* and *v* are the two-dimensional coordinates of the key point. $f_{x}$ and $f_{y}$ are the focal lengths of the camera. $u_{0}$ and $v_{0}$ are the coordinates of the principal point of the camera. The rotation matrix *R* and the translation matrix *T* are the extrinsic parameters of the camera, and *X*, *Y*, and *Z* are the three-dimensional spatial coordinates.

#### 2.7.3. Estimating on Fish Body Length and Width

The key points of the fish body are detected by the FishKP-YOLOv11 key point detection model, providing the two-dimensional coordinate information for the four key points. By combining the depth information of these key points with the internal and external parameters of the camera, the four key points are mapped from the 2D image to 3D space. The actual length (L) and width (W) of the fish body are then estimated using the three-dimensional Euclidean distance. The formulas for estimating the length and width are presented in Equations (2) and (3):

(2) $$L=\sqrt{{\left(X_{mouth}-X_{tail}\right)}^{2}+{\left(Y_{mouth}-Y_{tail}\right)}^{2}+{\left(Z_{mouth}-Z_{tail}\right)}^{2}},$$

in which $X_{mouth}$, $Y_{mouth}$, and $Z_{mouth}$ are the three-dimensional spatial coordinates of the key points of the Head, and $X_{tail}$, $Y_{tail}$, and $Z_{tail}$ are the three-dimensional spatial coordinates of the key points of the tail.

(3) $$W=\sqrt{{\left(X_{top}-X_{bottom}\right)}^{2}+{\left(Y_{top}-Y_{bottom}\right)}^{2}+{\left(Z_{top}-Z_{bottom}\right)}^{2}},$$

in which $X_{top}$, $Y_{top}$, and $Z_{top}$ are the three-dimensional spatial coordinates of the key points of the upper width, and $X_{bottom}$, $Y_{bottom}$, and $Z_{bottom}$ are the three-dimensional spatial coordinates of the key points of the lower width.

#### 2.7.4. Estimation Fish Body Mass

**Figure 12:**
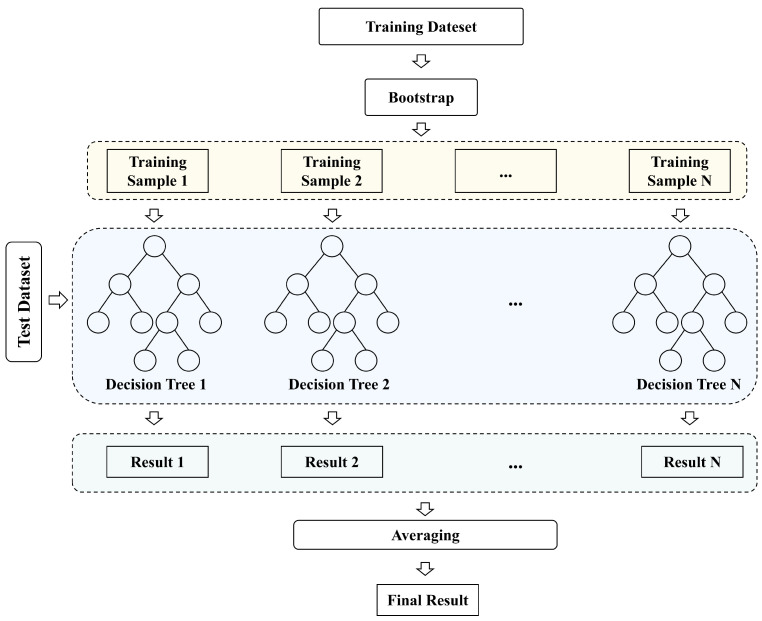
Random Forest model structure.

In estimating fish mass, this paper utilizes the Random Forest algorithm [50] to construct a nonlinear mapping model between body length, body width, and mass. As illustrated in Figure 12, the Random Forest algorithm efficiently models the nonlinear relationship between body length, body width, and mass by collaboratively predicting through multiple regression decision trees. During training, the Bootstrap Sampling method is applied to extract multiple training subsets, with each subset training a decision tree independently. In the node splitting process of the decision tree, the random subspace method is employed, where the optimal splitting attribute is determined solely from a randomly selected feature subset. This ensures both sample diversity and feature diversity. After training, the final prediction for the fish body mass is obtained by calculating the arithmetic mean of the outputs from all regression trees. Random Forest captures the complex nonlinear relationship between body length, body width, and mass through the diversity of base trees. Compared to traditional linear regression or single decision tree models, its prediction error is significantly reduced, and it exhibits a stronger ability to resist overfitting. Consequently, the Random Forest algorithm is chosen as the fish mass estimation model in this study.

### 2.8. Evaluation Indicators

#### 2.8.1. Evaluation Metrics for Object Detection and Key Point Detection

In the task of object detection, the Intersection over Union (IOU) defines the degree of overlap between the ground truth object and the predicted object. For key point detection, the evaluation metric used is the Object Key Point Similarity (OKS). The calculation of OKS is provided in Equation (4):

(4) $$OKS=\frac{\sum_{i}\exp\left(-\frac{d_{i}^{2}}{2s_{k}^{2}\sigma_{i}^{2}}\right)\cdot \delta \left(\nu_{i}>0\right)}{\sum_{i}\delta \left(\nu_{i}>0\right)},$$

where $d_{i}$ represents the Euclidean distance between the predicted and actual positions of the ith key point; $s_{k}$ is the area of the target bounding box; $\sigma_{i}$ is the standard deviation of the ith key point; $v_{i}$ is the visibility of the ith key point; $\delta_{i}$ is a function used to select visible points for calculation.

To evaluate the model’s performance, the mean average precision (mAP) is used for both object detection and key point detection. In object detection, precision and recall are calculated by setting an IOU threshold, whereas in key point detection, precision and recall are determined by setting an OKS threshold. The specific formulas for calculating mAP are shown in Equations (5)–(9):

(5) $$Precision=\frac{TP}{TP+FP},$$

(6) $$Recall=\frac{TP}{TP+FN}.$$

Here, TP represents the number of true positive samples, FP represents the number of false positive samples, and FN represents the number of false negative samples.

Average precision (AP) quantifies the model’s performance. Given the trade-off between precision and recall, the precision–recall (P–R) curve is used to represent AP. The larger the area enclosed by the P–R curve, the greater the AP, indicating better performance. The formula for calculating AP is shown in Equation (7):

(7) $$AP=\int_{0}^{1}P\left(r\right)dr.$$

To address the difficulty of calculating the integral, the interpolation method is employed for calculating AP. The formula for calculating AP using the interpolation method is shown in Equation (8):

(8) $$AP=\sum_{k=1}^{N}P\left(k\right)\Delta r\left(k\right).$$

mAP is obtained by averaging the AP values across all classes. When the number of classes is one, AP is equivalent to mAP. The formula for calculating mAP is shown in Equation (9), where C represents the number of classes.

(9) $$mAP=\frac{\sum_{i=1}^{c}AP_{i}}{C}$$

#### 2.8.2. Size and Mass Estimation Evaluation Indicators

To assess the accuracy of the mass estimation model, this study employs the mean absolute error (MAE), root mean square error (RMSE), and coefficient of determination (R^2^) for analysis. The specific formulas are shown in Equations (10)–(13):

(10) $$MAE=\frac{1}{m}\sum_{i=1}^{m}\left|x_{i}-y_{i}\right|,$$

(11) $$MSE=\frac{1}{m}\sum_{i=1}^{m}{\left(x_{i}-y_{i}\right)}^{2},$$

(12) $$RMSE=\sqrt{\frac{1}{m}\sum_{i=1}^{m}{\left(x_{i}-y_{i}\right)}^{2}},$$

(13) $$R^{2}=\frac{{\left[\sum_{i=1}^{m}\left(x_{i}-\bar{x}\right)\left(y_{i}-\bar{y}\right)\right]}^{2}}{\sum_{i=1}^{m}{\left(x_{i}-\bar{x}\right)}^{2}\sum_{i=1}^{n}{\left(y_{i}-\bar{y}\right)}^{2}}.$$

Specifically, $x_{i}$ represents the true value, $y_{i}$ denotes the predicted value, *m* is the number of fish, $\bar{x}$ is the mean of the true values, and $\bar{y}$ is the mean of the predicted values.

## 3. Experimental Results and Analysis

This chapter systematically presents and analyzes the experimental verification results of the proposed method. The experiments provide a comprehensive evaluation of the performance of the FishKP-YOLOv11 model and the fish size and mass estimation method, focusing on the following key aspects: (1) evaluation of the FishKP-YOLOv11 model’s performance through comparisons with advanced key point models, ablation experiments, and multi-environment robustness tests; (2) assessment of the effectiveness of the fish size and mass estimation method based on key point detection, including the estimation of fish size and mass across various body types and environments.

### 3.1. Key Point Detection

#### 3.1.1. Comparison with the State of the Art

To verify the effectiveness of the FishKP-YOLOv11 model proposed in this paper, it was compared with several YOLO series models that have achieved the best balance between accuracy and speed, including YOLOv5 [51], YOLOv6 [52], YOLOv7 [53], YOLOv8 [54], YOLOv9 [55], YOLOv10 [56], YOLOv11, and YOLOv12 [57]. The results are presented in Table 3. The mAP50 for object detection of FishKP-YOLOv11 is 91.8%, and the mAP50-95 is 73.5%, surpassing all the comparison models. Additionally, the model parameter is only 2.9M. FishKP-YOLOv11 has a comparable model parameter to the lightest models in each YOLO series, with the mAP50 for object detection being 3.6%, 7.3%, 3.9%, 3.5%, 3.0%, 4.3%, and 4.0% higher than YOLOv5n, YOLOv6n, YOLOv8n, YOLOv9t, YOLOv10n, YOLOv11n, and YOLOv12n, respectively. Compared to the second-best YOLOv10m, the mAP50 of FishKP-YOLOv11 for object detection is 1.4% higher, while the parameter size is only 16% that of YOLOv10m. This indicates that the lightweight FishKP-YOLOv11 model can maintain high-precision localization capabilities across different IOU thresholds. Especially in complex underwater scenarios, its detection ability significantly outperforms traditional YOLO models and the latest YOLO versions. For key point detection, the mAP50 and mAP50-95 of FishKP-YOLOv11 are 91.7% and 91.3%, respectively, again outperforming all comparison models. Compared to the lightest YOLOv5n, YOLOv6n, YOLOv8n, YOLOv9t, YOLOv10n, YOLOv11n, and YOLOv12n, the key point detection mAP50 is 3.3%, 7.3%, 4.0%, 3.8%, 3.0%, 4.2%, and 4.1% higher, respectively. Compared to the second-best YOLOv10m, the key point detection mAP50 of FishKP-YOLOv11 is 1.4% higher, demonstrating its outstanding performance advantages. The experiments confirm that FishKP-YOLOv11’s superior performance and lightweight model parameters enable deployment on resource-constrained devices in real aquaculture environments, facilitating efficient and accurate fish key point detection and providing solid technical support for the intelligentization of aquaculture.

#### 3.1.2. Ablation Study

To analyze the impact of each module on the model’s performance, ablation experiments were conducted on the FishKP-YOLOv11 model using the key point detection dataset. The results are shown in Table 4. FishKP-YOLOv11 uses YOLOv11n as the baseline. After incorporating the HGCBlock module, which includes the HCEBlock module as the main feature extraction component of the model, the mAP50 for object detection and key point detection improved by 1.6% and 1.3%, respectively, while the mAP50-95 increased by 1.3% and 1.4%, respectively. The number of parameters increased by 0.2 M. To further enhance model performance while reducing the number of parameters, the FPN feature fusion method was introduced in the Neck network component. This resulted in a 1.3% improvement in mAP50 for object detection and a 1.6% improvement for key point detection. The mAP50-95 also improved by 2% for object detection and 1.7% for key point detection. The number of parameters decreased by 0.6 M. To further strengthen the model’s feature extraction capability, the GDCBlock module was added to the HGCBlock module to adjust the weight features. This addition led to a 0.9% improvement in mAP50 for both object detection and key point detection, with mAP50-95 improving by 2.2% and 1%, respectively. The number of model parameters increased by 1.1 M. Finally, to enhance the model’s feature analysis capacity and reduce model parameters, a double-layer depth convolution was introduced in the class branch and bounding box branch of the Head network, deepening the network while reducing the model parameters. This change resulted in a 0.5% improvement in mAP50 for object detection and a 0.4% improvement for key point detection, with mAP50-95 improving by 0.2% for object detection and 0.4% for key point detection. The number of parameters decreased by 0.3 M. The experiments demonstrate that each module positively impacts the model’s performance, and the proposed model meets the performance and parameter requirements for actual aquaculture scenarios.

#### 3.1.3. The Performance of Key Point Detection Models in Different Environments

To verify the model’s performance under various environmental conditions, this paper tests the FishKP-YOLOv11 model in five distinct environments: turbid and low stocking density, turbid and high stocking density, clear and low stocking density, clear and high stocking density, and low-light conditions. The results are shown in Table 5. The mAP50 for object detection and key point detection in both turbid and low stocking density environments, as well as clear and low stocking density environments, reaches 95.7%, indicating that the model can effectively cope with different water qualities. In the turbid and high-density stocking environment, as well as the clear and high-density stocking environment, the mAP50 for both object detection and key point detection decreases slightly but remains around 91%. It demonstrates that the model not only performs well under varying water quality conditions but also effectively addresses challenges associated with high-density scenarios. In summary, FishKP-YOLOv11 can maintain high detection accuracy across different water qualities, lighting conditions, and stocking densities, effectively handling various complex situations in actual aquaculture scenarios.

**Figure 13:**
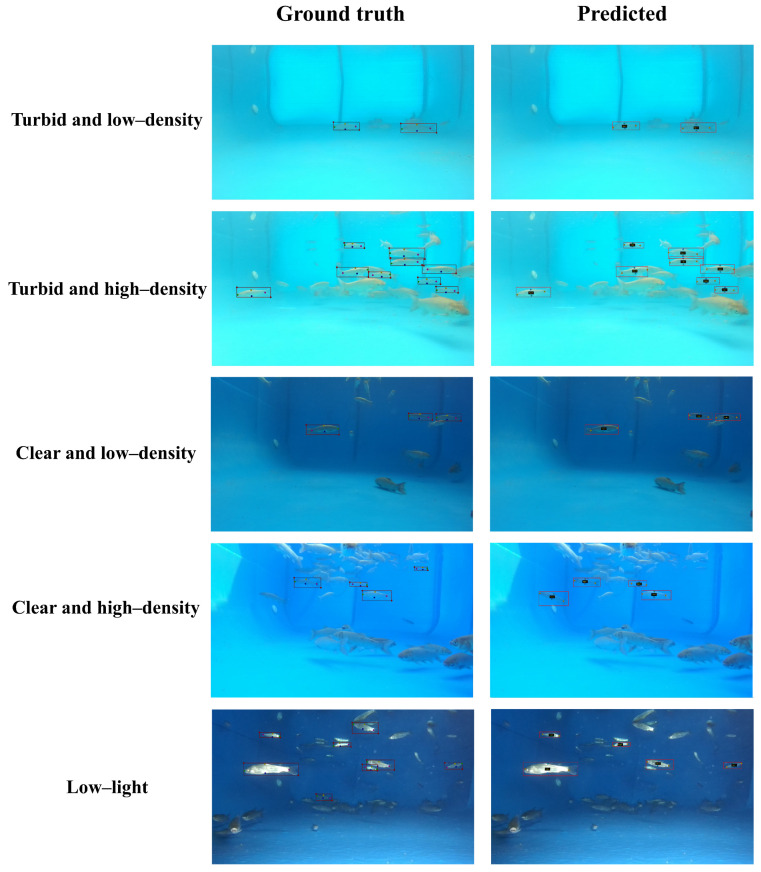
Detection performance of the model in different environments.

To better observe the model’s performance, the detection results of the model in different environments are presented in Figure 13. In low-density aquaculture environments, the model can accurately identify the target fish and mark its key points, with minimal errors even in turbid water. In high-density aquaculture environments and low-light conditions, the model may exhibit a few misjudgments; however, its overall recognition accuracy remains high. The key point predictions are consistently accurate across various environmental conditions, highlighting the model’s stability and reliability in complex environments.

### 3.2. Fish Size and Mass Estimation

#### 3.2.1. Size Estimation

In this study, the body length and body width of grass carp were calculated using the three-dimensional Euclidean distance between key points. To verify the accuracy of this size estimation method, four groups of grass carp samples with varying specifications were selected for the experiment. After measuring their actual body length, body width, and mass, three images of each fish were randomly selected, and the proposed method was applied for size estimation. The results are presented in Table 6 and Table 7. The average absolute error (MAE) between the predicted and actual body length was 0.35, while the MAE between the predicted and actual body width was 0.1. As the size of the grass carp decreased, the prediction error for the length also decreased, while the prediction error for the width remained relatively stable and accurate. This study showed that the prediction of body width was less influenced by the fish’s posture, leading to stable and accurate prediction results. In contrast, the prediction of body length was more affected by posture, and even slight bending could introduce errors. Despite this, the body length information calculated using the key point detection model still maintains a high level of accuracy.

**Figure 14:**
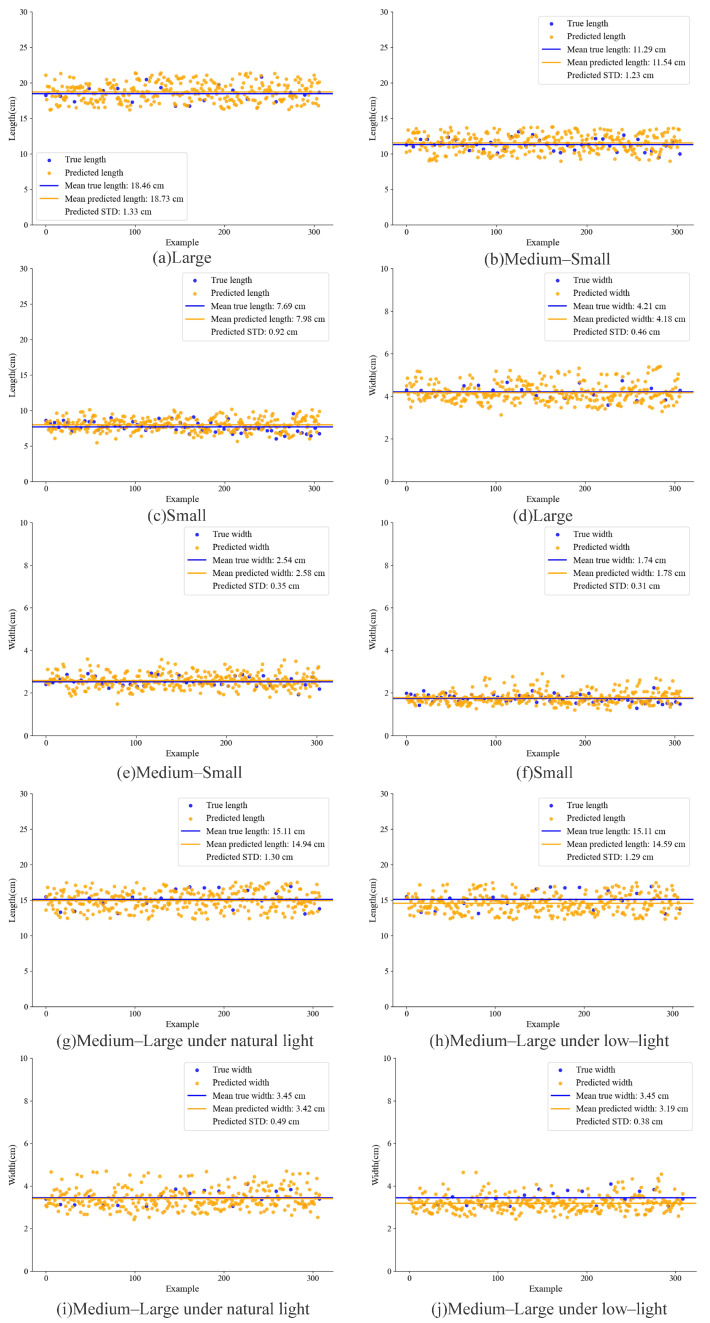
Comparison of actual mean sizes and predicted mean sizes was conducted for fish across different size groups. Panels (**a**–**g**,**i**) illustrate the comparisons between actual and predicted mean sizes of four fish school–size categories under natural lighting. Panels (**g**–**j**) illustrate the comparisons between actual and predicted mean sizes of medium–large fish under varying lighting conditions.

To further evaluate the proposed size estimation method on fish schools, four school-size categories were selected: large, medium–large, medium–small, and small. The results are presented in Figure 14. Figure 14a–c,g compare the predicted lengths with the actual lengths for the four size categories, and Figure 14d–f,i compare the predicted widths with the actual widths. The absolute errors between predicted and measured average lengths were 0.27 cm, 0.25 cm, 0.29 cm, and 0.17 cm for the large, medium–large, medium–small, and small groups, respectively. The corresponding predicted standard deviations were 1.33 cm, 1.30 cm, 1.23 cm, and 0.92 cm. The absolute errors between predicted and measured average widths were 0.03 cm, 0.04 cm, 0.04 cm, and 0.03 cm, respectively, with standard deviations of 0.46 cm, 0.49 cm, 0.35 cm, and 0.31 cm. Overall, the results indicate that the proposed method can accurately estimate population-level fish dimensions, supporting its use for precise measurements in aquaculture.

To investigate the influence of lighting on size estimation accuracy, the estimation results of fish groups classified as medium–large were compared between natural daytime illumination and low-light nighttime conditions. The results appear in Figure 14. Figure 14g compares the predicted and actual mean lengths under natural light; the absolute error was 0.17 cm. Figure 14h shows the same comparison under nighttime low-light conditions; the absolute error was 0.52 cm. Figure 14i compares the predicted and actual mean widths under natural light; the absolute error was 0.03 cm. Figure 14j presents the width comparison under nighttime low-light; the absolute error was 0.26 cm. The results indicate that increased image noise and loss of body detail under low-light conditions raise prediction errors at night relative to daytime conditions. Nevertheless, the method maintains a high level of prediction accuracy even in low-light scenarios, which further validates its robustness and practical applicability.

#### 3.2.2. Mass Prediction

The accuracy of the Random Forest algorithm for predicting fish body mass was evaluated by comparison with several algorithms, including LASSO [58], Ridge [59], linear [60], ExtraTree [61], AdaBoost [62], DecisionTree [63], KNN [64], Bagging [65], and GradientBoost [66]. During the experiment, 80% of the data were used for training and 20% for testing. The results are presented in Table 8. Random Forest outperformed the other methods across the evaluated metrics. It achieved a mean absolute error (MAE) of 1.2737, a mean squared error (MSE) of 6.7184, a root mean squared error (RMSE) of 2.592, and an R^2^ of 0.9968. Compared with alternative algorithms, Random Forest demonstrated greater stability and higher accuracy in estimating fish body mass. When fish body length and width were used as predictors, all tested methods yielded MAE values below 10 and R^2^ values above 0.9. These results indicate a strong correlation between fish length, width, and mass and support the validity of using length and width to predict mass.

To validate the practical effectiveness of the Random Forest-based mass prediction method, size estimation results from four grass carp groups of different body sizes were used as inputs to predict mass. Predicted masses were then compared with the corresponding measured masses. As shown in Table 9, the mean absolute error between predicted and actual mass was 2.7 g, indicating high prediction accuracy. Prediction error decreased as fish size decreased; the smallest size class exhibited an average absolute error of 0.9 g. These results confirm the Random Forest model’s accuracy and practicality for fish mass estimation.

**Figure 15:**
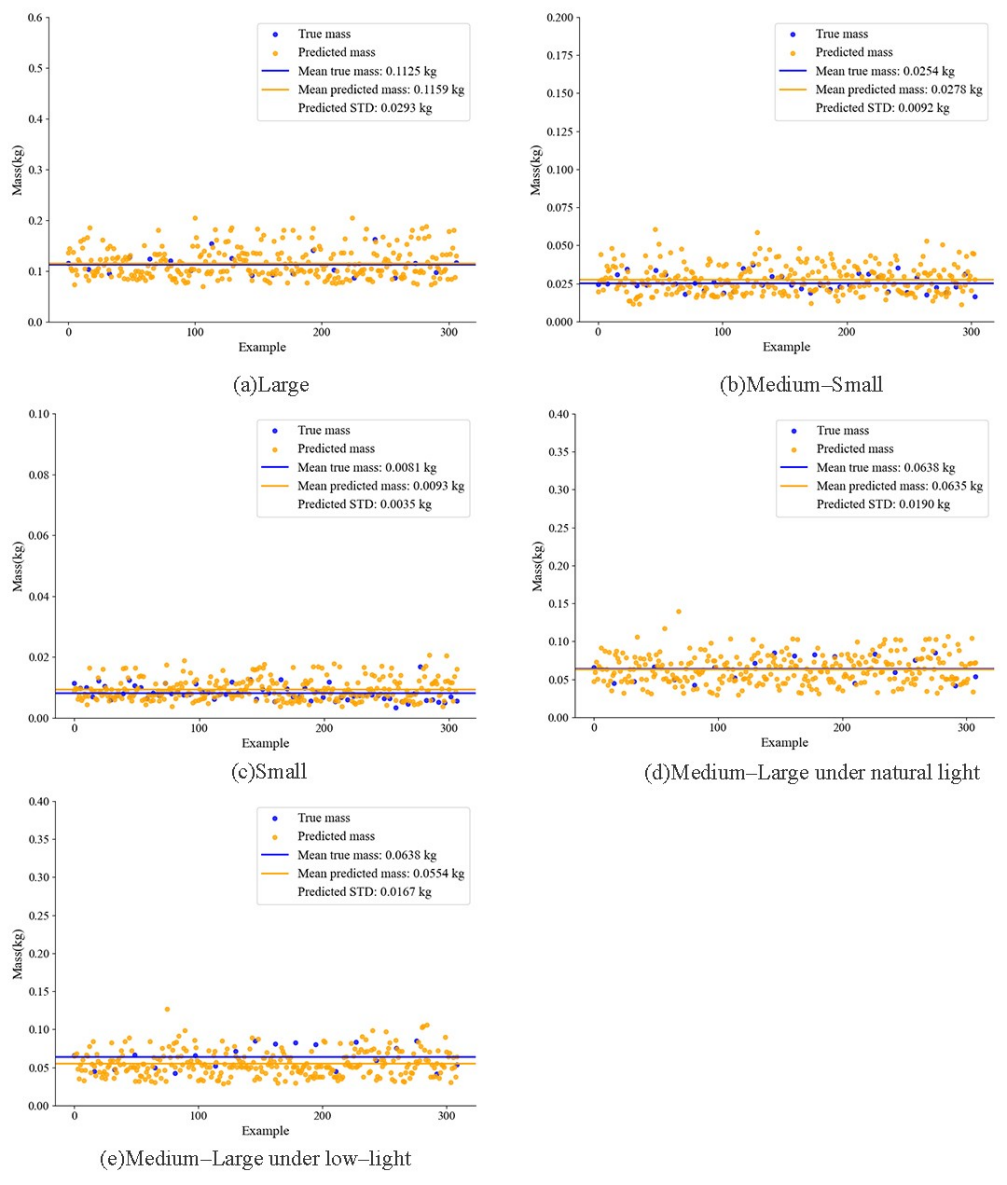
Comparison of actual and predicted average weights was performed for fish across different size groups. Panels (**a**–**d**) illustrate the comparisons between actual and predicted mean mass of four fish school–size categories under natural lighting. Panels (**d**,**e**) present the results for medium–large fish under varying lighting conditions.

The applicability of a quality prediction method based on Random Forest for fish groups was validated using the size-prediction results for four body size categories (large, medium–large, medium–small, and small) shown in Figure 14 to predict average weights. The predicted averages were compared with the measured averages, and the comparisons are presented in Figure 15. For large fish, the predicted average mass differed from the measured average by 3.4 g. For medium–large fish, the difference was 0.3 g. For medium–small fish, the difference was 2.4 g. For small fish, the difference was 1.2 g. The corresponding standard deviations were 29.3 g, 19.0 g, 9.2 g, and 3.5 g, respectively. These findings indicate that the Random Forest-based method accurately predicts average mass for groups of different sizes, with prediction error decreasing as fish size decreases. Further analysis identifies two contributing factors. First, size estimation is more accurate for smaller fish, which yields more precise mass predictions. Second, as grass carp grow, morphological changes occur not only in length and width but also in body thickness. These additional variations increase prediction complexity and consequently reduce model accuracy. Together, these factors influence the performance of the mass prediction model. In summary, the Random Forest mass prediction method demonstrates strong applicability and accuracy across the four examined size classes, supporting its use for precise management at different aquaculture stages.

To evaluate the effect of illumination on mass prediction performance, the size estimation results under natural light and low-light conditions in Figure 14g–j were used to predict the population mean mass and were compared with measured averages. The results, shown in Figure 15d,e, indicate that the predicted mean mass under natural light differed from the measured mean by 0.3 g. Under low-light conditions, the predicted mean mass differed from the measured mean by 8.4 g. These findings show that lighting has a substantial impact on prediction accuracy. Reduced visibility and loss of surface texture under low-light, together with increased image noise, substantially degrade feature quality and thereby increase prediction error. Nevertheless, the prediction error under low-light remained within an acceptable range for the intended application. This outcome suggests that the Random Forest-based mass prediction method retains a degree of robustness across varying illumination conditions and can support stable mass estimation in the actual aquaculture environment, thereby offering practical value for fishery management.

**Figure 16:**
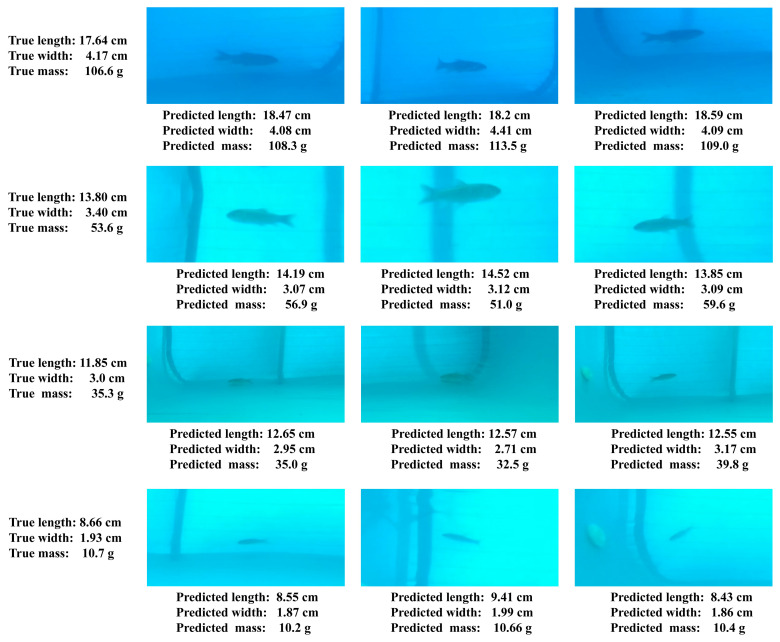
Results of size and mass prediction for grass carp in the case of blurred images.

Variable water quality and illumination in actual aquaculture often produce blurred images. To evaluate the proposed size and mass estimation method under such conditions, blurred images of four grass carp representing different size classes were used for estimation. The results are presented in Figure 16. Even when fish bodies were blurred and surface texture was lost, length estimation errors for all size classes remained within 0.9 cm, width estimation errors remained within 0.4 cm, and mass prediction errors remained within 7 g. These findings demonstrate that the proposed method maintains high accuracy under image blurring conditions, ensures stable performance in challenging water quality and lighting environments, and provides a reliable monitoring tool for aquaculture.

## 4. Discussion

### 4.1. The Impact of Improvements on the Model

This study compares performance differences among various YOLO versions and among models with different parameter sizes within each version. As shown in Table 3, across the YOLO series, the second-lightest models consistently outperform the minimal-parameter versions. Increasing model width and depth beyond this point does not produce sustained gains. These findings indicate that, in complex underwater scenarios, scaling models by widening or deepening alone does not improve feature learning. Such scaling can degrade generalization through overfitting and introduce redundant computations. Existing YOLO variants that are not specifically adapted to underwater conditions therefore struggle to learn practical features in these environments. The performance gain of FishKP-YOLOv11 arises from the combined optimization of an underwater-specific feature extraction module (HGCBlock, comprising HCEBlock and GDCBlock), the Feature Pyramid Network (FPN), and an enhanced Head network. This combined design attains superior mAP while keeping parameter counts comparable to the lightest versions of other YOLO variants.

As shown in Table 4, ablation experiments on FishKP-YOLOv11 show that improvements to each component increase overall performance. The HCEBlock module implements a dual-branch hybrid convolution strategy. The deep convolution branch extracts semantic features, while the shallow convolution branch preserves fine details. This arrangement captures key fish features in turbid and low-light conditions and prevents loss of edge features caused by water turbidity. The integration of a Feature Pyramid Network (FPN) reduces information overlap and redundant feature computations that arise from repeated interactions among multi-directional, multi-scale features. The FPN also enables robust processing of low-light, high-noise underwater images, thereby improving detection accuracy. The GDCBlock module implements a gated dynamic weighting mechanism together with a residual fusion strategy. These mechanisms strengthen the representation of crucial target-body features and preserve detailed information, which improves the model’s ability to distinguish subtle differences among fish bodies. Significantly, the HCEBlock, FPN, and GDCBlock yield significant performance enhancements. Finally, lightweight modifications to the Head network reduced the number of model parameters without sacrificing performance. The enhanced ability to distinguish target fish from background fish further contributes to the overall improvement of the model.

### 4.2. The Impact of Different Underwater Environments on Model Performance

Different underwater environments affect image quality, and image quality in turn affects key point detection accuracy. In turbid water, white flocculent matter obscures the scene, which degrades fish body imaging and impairs key point detection. The HGCBlock module in FishKP-YOLOv11 provides strong feature extraction capability. As shown in Table 5, the model attains similar mAP scores in both clear and turbid water. This result demonstrates the model’s ability to mitigate feature extraction challenges induced by water turbidity. In low-density farming environments, fish distribution is sparse, and interference from free-swimming individuals is minimal. By contrast, high-density farming environments present increased overlap and occlusion, greater pose variability, and more frequent confusion between target and background fish bodies. These conditions together reduce model performance. The GDCBlock module and the Head network improve the model’s ability to recognize subtle differences in fish bodies. Consequently, the model achieved accuracies of 91.1% and 90.9% in high-density farming environments. This result demonstrates effective handling of the challenges to high-density aquaculture.

In low-light environments, image noise surges, fish body features are severely lost, and issues such as color and white balance shifts further complicate the model’s ability to extract practical features. By virtue of the FPN in FishKP-YOLOv11, the feature fusion stage avoids excessive feature interactions that cause information overlap and redundant feature computations. Simultaneously, it enables the model to leverage shallow-layer features to extract more detailed information, effectively addressing the challenge of feature extraction in low-light conditions. As shown in Figure 14d,e,i,j, medium-to-large fish populations were selected for comparison under natural lighting and nighttime low-light conditions. The average length estimation error under natural lighting was less than under low-light conditions. This is because, under natural lighting, key point detection accuracy is higher, and stereo matching provides more precise fish depth estimation, resulting in a smaller average length estimation error. However, even under low-light conditions, the average length estimation error was only 0.52 cm. Since mass estimation accuracy depends on size estimation accuracy, as shown in Figure 15, mass estimation for medium-to-large fish under natural light and nighttime low-light conditions followed the same pattern. The average mass estimation error under low-light conditions was 8.4 g, which remains within an acceptable range.

### 4.3. Analysis of Estimation Errors for Fish of Different Sizes

Table 6 shows that estimation errors increase with the growth of fish length. Analysis of swimming behavior indicates that larger fish have longer bodies and, consequently, are more prone to slight bending during locomotion. Because length estimation depends on body posture, even minor bending produces measurable errors. In contrast, estimation errors for fish width do not display a significant increase with size. Table 7 shows that width errors remain relatively consistent across size categories. During swimming, the trunk bends less in the lateral direction, which enables more precise width estimation. Overall, the mean absolute error (MAE) for length estimation was 0.35 cm, while the MAE for width estimation was 0.10 cm, indicating high estimation accuracy. Since fish mass estimation is derived from length and width measurements, its error trend follows that of length estimation. Table 9 shows that the estimation error of fish mass exhibits an increasing trend with the increasing trend of fish size. The overall MAE for mass estimation was 2.7 g, indicating high estimation accuracy.

As shown in Figure 14, the estimation of length and width across fish schools of different sizes reveals that larger fish generate greater standard deviations in both parameters. As illustrated in Figure 15, the estimated mass derived from these length and width values exhibits a similar trend, with larger fish producing greater standard deviations in mass estimation. The maximum estimation errors of the average length and width were 0.29 cm and 0.04 cm, respectively, whereas the maximum error of the average mass was 3.4 g. Although the estimation errors for size and mass increase with the augmentation of fish dimensions, they remain confined within a narrow range and meet the practical requirements of aquaculture applications.

### 4.4. The Practical Significance of Real-Time Edge Deployment

In practical aquaculture scenarios, the deployment of high-performance hardware is infeasible. The primary reason lies in economic constraints, as such equipment typically incurs high costs. Furthermore, objective limitations, particularly in deep-sea aquaculture, impose additional challenges, such as restricted energy supply and unstable communication connectivity, which make cloud-based deployment impractical in aquaculture environments. In contrast, lightweight models can be deployed on resource-constrained devices to support real-time edge processing. The FishKP-YOLOv11 model proposed in this study, containing only 2.9 M parameters, illustrates the feasibility of real-time inference under such conditions. When deployed locally on resource-constrained devices, the model enables on-site processing without reliance on cloud transmission, thereby reducing latency, improving response speed, and lowering network bandwidth requirements. These advantages are critical for the real-time monitoring of fish growth, timely adjustment of feeding strategies, and the early detection of abnormal behaviors.

### 4.5. Limitations of This Study

The proposed method demonstrates high accuracy in complex aquaculture environments, but several limitations remain that require further investigation in future research. Firstly, the dataset in this study was constructed according to the principle of labeling only fish that are parallel to the imaging plane and free from key point occlusion or severe body curvature. This strategy ensures high-quality training data but also introduces dataset bias and restricts model generalization. In practical aquaculture environments, fish often tilt relative to the imaging plane, undergo partial occlusion, or display slight bending due to swimming and collision avoidance. Because such cases are not included in the dataset, the model’s generalization ability may be limited. Secondly, the proposed size and mass estimation method was validated only on grass carp, without testing across other fish species. The effectiveness of this method in estimating the size and mass of diverse fish species remains uncertain. Finally, the robustness of this method has not been verified in actual aquaculture systems, such as ponds, net pens, or offshore farms. These environments contain suspended solids, algae, and organic matter. Moreover biofouling on equipment, low water transparency, and unstable lighting further complicate the imaging conditions. Therefore, validation of the method’s robustness under these more challenging scenarios is necessary.

## 5. Conclusions

Estimating fish size and mass in actual aquaculture is challenged by complex water quality, variable illumination, and diverse fish postures. The proposed method, FishKP-YOLOv11, is more suitable for the high-density aquaculture environment. A grass carp dataset was constructed that covers multiple water qualities, lighting conditions, and stocking densities. To overcome the problem of extracting fish body features in complex underwater scenes, the key point detection model was proposed, the core extractor of which is composed of the HCEBlock and GDCBlock. The Feature Pyramid Network (FPN) was also incorporated. In addition, the Head network’s classification and bounding box regression branches, which support key point detection, were redesigned. Compared with the baseline model YOLOv11, FishKP-YOLOv11 achieved a 4.3% improvement in object detection mAP50 and a 4.2% improvement in key point detection mAP50, reaching 91.8% and 91.7%, respectively. The model also outperformed multiple versions of YOLOv5, YOLOv6, YOLOv8, YOLOv9, YOLOv10, YOLOv11, and YOLOv12. A size-computation method combined detected key points with stereo vision technology to estimate body length and width. On the grass carp dataset, the mean absolute error (MAE) between estimated and measured body length was 0.35 cm. The MAE for body width was 0.1 cm. Average sizes for groups of different sizes and for groups under varying illumination were also estimated, and the estimated means closely matched the measured means. A Random Forest-based mass prediction model was then developed. In practical mass prediction tests, the mean absolute error between predicted and actual mass was 2.7 g. Predicted mean masses for groups of different sizes and under different lighting conditions were close to the corresponding measured means. In summary, the proposed method for fish size and mass prediction demonstrated strong performance and robustness in complex tank environments and shows potential for practical application in aquaculture, providing an accurate approach for size and mass monitoring.

To improve the applicability of the fish size and mass estimation framework in practical aquaculture, future research will focus on several directions. Firstly, full-angle key point annotation methods will be investigated in combination with image enhancement techniques. This approach aims to reduce size estimation errors caused by curved fish postures and to strengthen the model’s generalization to complex body poses. Secondly, the framework will be extended to multi-species scenarios. In this process, physiological and structural differences among fish species will be incorporated to establish more universal size and mass prediction models. Moreover, although strong correlations exist between fish length or width and mass, these relationships tend to weaken as fish grow. Therefore, future work will integrate additional morphological features, such as the fish’s girth, to improve estimation accuracy and robustness. Finally, the framework will be validated through large-scale trials and long-term verification in multiple aquaculture systems, ensuring stability and supporting future industrial applications. 

## Figures and Tables

**Table 1:** Number of images included in the dataset for different light conditions, water quality, stocking density, and fish size.

Type	Number
Low-density and turbid water	316
High-density and turbid water quality	260
Low-density and clear water quality	260
High-density and clear water quality	258
Low-light at night	260
Large	259
Medium–large	258
Medium–large at night	265
Medium–small	275
Small	263

**Table 2:** Experimental environment.

Configuration	Parameter
CPU	AMD EPYC 7402 24-Core
GPU	RTX3090
Operating system	Ubuntu 22.04
Deep learning framework	Pytorch 2.5.0
Programming language	Python 3.10.0
CUDA	11.8

**Table 3:** Results of comparisons with different YOLO versions.

Model	Object mAP50 (%)	Object mAP50-95 (%)	Pose mAP50 (%)	Pose mAP50-95 (%)	Parameters (M)
YOLOv5n	88.2	68.4	88.4	87.5	2.4
YOLOv5s	89.5	69.0	89.4	88.9	8.9
YOLOv5m	89.2	70.2	89.3	88.8	24.5
YOLOv5l	89.3	71.8	89.3	88.9	51.7
YOLOv5x	89.4	71.3	89.3	88.9	94.3
YOLOv6n	84.5	58.1	84.4	82.2	4.0
YOLOv6s	86.6	63.6	87.0	85.6	15.6
YOLOv6m	77.6	51.7	77.0	74.1	49.7
YOLOv6l	77.4	50.7	78.3	75.4	106.0
YOLOv6x	82.2	56.2	82.2	79.8	165.3
YOLOv8n	87.9	66.7	87.7	86.9	2.9
YOLOv8s	88.2	69.3	88.1	87.5	10.8
YOLOv8m	88.7	71.2	88.7	88.4	25.1
YOLOv8l	88.1	70.3	88.0	87.6	42.3
YOLOv8x	88.8	71.4	88.8	88.4	66.2
YOLOv9t	88.3	65.7	87.9	87.2	1.9
YOLOv9s	89.7	69.1	89.6	88.9	7.0
YOLOv9m	88.0	71.1	88.0	87.7	19.7
YOLOv9c	89.3	68.7	89.4	88.7	24.9
YOLOv9e	87.6	70.9	87.6	87.3	55.5
YOLOv10n	88.8	66.7	88.7	87.7	2.5
YOLOv10s	90.1	70.4	90.1	89.5	8.3
YOLOv10m	90.4	72.1	90.3	90.0	17.6
YOLOv10b	89.5	71.9	89.4	89.1	22.9
YOLOv10l	88.9	68.9	88.8	88.1	27.9
YOLOv10x	87.3	69.1	87.3	86.9	35.5
YOLOv11n	87.5	67.8	87.5	86.8	2.5
YOLOv11s	89.4	70.8	89.3	88.8	9.2
YOLOv11m	89.1	71.3	89.0	88.5	19.9
YOLOv11l	89.9	70.5	89.8	89.4	24.9
YOLOv11x	88.7	72.7	88.8	88.5	56.0
YOLOv12n	87.8	65.2	87.6	86.6	2.5
YOLOv12s	89.4	69.8	89.5	88.8	9.0
YOLOv12m	90.0	72.2	89.9	89.5	19.9
YOLOv12l	89.6	71.3	89.6	89.1	25.9
YOLOv12x	89.4	72.4	89.5	89.1	58.1
FishKP-YOLOv11	91.8	73.5	91.7	91.3	2.9

**Table 4:** Evaluation results of ablation experiments on the FishKP-YOLOv11 architecture with YOLOv11n as the baseline—progressive adding of the HCEBlock, FPN, GDCBlock, and redesigned Head.

Method	Object mAP50 (%)	Object mAP50-95 (%)	Pose mAP50 (%)	Pose mAP50-95 (%)	Parameters (M)
Baseline	87.5	67.8	87.5	86.8	2.5
+HCEBlock	89.1	69.1	88.8	88.2	2.7
+FPN	90.4	71.1	90.4	89.9	2.1
+GDCBlock	91.3	73.3	91.3	90.9	3.2
+Head	91.8	73.5	91.7	91.3	2.9

**Table 5:** Performance of the model in different underwater environments and at different stocking densities.

Environment	Object mAP50 (%)	Object mAP50-95 (%)	Pose mAP50 (%)	Pose mAP50-95 (%)
turbid and low stocking density	95.7	79.6	95.7	95.7
turbid and high stocking density	91.1	69.5	91.2	90.6
clear and low stocking density	95.7	82.3	95.7	95.3
clear and high stocking density	90.9	72.7	90.8	90.2
low-light conditions	88.1	66.1	87.8	87.4

**Table 6:** Estimation results of the lengths of grass carp of different sizes.

Type	Number	Real	P-1	P-2	P-3	M-1	M-2	M-3
L	1	17.65	18.2	18.47	18.03	0.55	0.82	0.38
	2	16.98	17.95	17.83	17.37	0.97	0.85	0.39
	3	17.71	18.59	17.69	17.9	0.88	0.02	0.19
	4	17.26	18.08	17.83	17.63	0.82	0.57	0.37
	5	18.53	18.92	18.42	19.14	0.39	0.11	0.61
M–L	6	15.46	15.67	14.95	15.59	0.21	0.51	0.13
	7	13.30	13.13	13.57	13.84	0.17	0.27	0.54
	8	14.56	14.91	14.15	14.3	0.35	0.41	0.26
	9	13.15	12.79	13.55	12.72	0.36	0.4	0.43
	10	15.40	15.58	15.38	15.53	0.18	0.02	0.13
M–S	11	11.85	12.56	12.65	12.76	0.71	0.8	0.92
	12	12.35	12.44	13.17	12.77	0.09	0.82	0.42
	13	11.02	11.05	11.66	10.86	0.03	0.64	0.16
	14	10.03	10.53	10.39	10.4	0.50	0.36	0.37
	15	11.31	11.46	11.98	11.38	0.15	0.67	0.07
S	16	8.66	8.55	9.0	8.45	0.11	0.34	0.21
	17	8.17	8.13	8.62	8.11	0.04	0.45	0.06
	18	9.01	9.21	9.17	9.07	0.20	0.16	0.06
	19	8.86	8.87	9.06	9.11	0.01	0.20	0.25
	20	9.01	9.08	8.92	8.99	0.07	0.09	0.02

L, M–L, M–S, and S represent grass carp of large, medium–large, medium–small, and small sizes, respectively. “Real” denotes the actual length, “P” denotes the predicted value, and “M” denotes the mean absolute error. All values are expressed in centimeters.

**Table 7:** Estimation results of the widths of grass carp of different sizes.

Type	Number	Real	P-1	P-2	P-3	M-1	M-2	M-3
L	1	4.17	4.41	4.08	4.27	0.24	0.09	0.10
	2	4.03	4.1	3.82	3.8	0.07	0.21	0.23
	3	3.90	3.8	3.89	3.84	0.1	0.01	0.06
	4	4.18	4.15	3.98	4.4	0.03	0.20	0.22
	5	4.36	4.23	4.36	4.31	0.13	0	0.05
M–L	6	3.397	3.32	3.53	3.3	0.08	0.13	0.10
	7	3.13	3.01	3.26	3.02	0.12	0.13	0.11
	8	3.10	3.23	3.03	3.17	0.13	0.07	0.08
	9	3.09	2.96	3.14	3.03	0.13	0.05	0.06
	10	3.42	3.53	3.6	3.37	0.11	0.18	0.05
M–S	11	3.0	2.77	2.95	2.9	0.23	0.05	0.10
	12	2.85	2.66	2.75	2.91	0.19	0.10	0.06
	13	2.68	2.7	2.62	2.5	0.02	0.06	0.18
	14	2.54	2.54	2.49	2.38	0	0.05	0.16
	15	2.49	2.49	2.58	2.56	0	0.09	0.07
S	16	1.93	1.87	1.86	1.8	0.06	0.07	0.13
	17	1.87	1.82	1.91	1.74	0.05	0.04	0.13
	18	1.95	1.92	2.01	1.89	0.03	0.06	0.06
	19	1.91	1.78	1.89	1.91	0.13	0.02	0
	20	1.98	1.87	1.83	2.15	0.11	0.15	0.17

L, M–L, M–S, and S represent grass carp of large, medium–large, medium–small, and small sizes, respectively. “Real” denotes the actual width, “P” denotes the predicted value, and “M” denotes the mean absolute error. All values are expressed in centimeters.

**Table 8:** Comparison results of different methods for predicting fish mass.

Model	MAE	MSE	RMSE	R^2^
LASSO	9.4860	124.4608	11.1562	0.9487
Ridge	9.1819	131.9871	11.4886	0.9400
Linear	8.7127	127.1564	11.2764	0.9394
ExtraTree	4.1029	44.5574	6.6751	0.9797
AdaBoost	3.1835	18.5412	4.3059	0.9950
DecisionTree	2.8441	21.1238	4.5961	0.9892
KNN	2.6382	23.3943	4.8368	0.9886
Bagging	2.5941	19.5434	4.4208	0.9915
GradientBoost	2.1987	12.7161	3.5660	0.9956
RandomForest	1.2737	6.7184	2.5920	0.9968

**Table 9:** Estimation results of the mass of grass carp of different sizes.

Type	Number	Real	P-1	P-2	P-3	M-1	M-2	M-3
L	1	106.6	113.5	108.3	107.6	6.9	1.7	1.0
	2	95.7	105.5	89.4	87.5	9.8	6.3	8.2
	3	90.6	92.0	93.5	93.1	1.4	2.9	2.5
	4	103.2	108.6	94.4	111.5	5.4	8.8	8.3
	5	124.3	117.5	116.7	120.1	6.8	7.6	4.2
M–L	6	65.6	64.2	66.4	62.6	1.4	0.8	3.0
	7	44.5	42.8	49.3	44.9	1.7	4.8	0.4
	8	49.9	53.2	47.3	49.7	3.3	2.6	0.2
	9	42.6	35.0	45.4	38.7	7.6	2.8	3.9
	10	65.3	67.8	72.0	63.5	2.5	6.7	1.8
M–S	11	35.3	32.9	35.0	34.5	2.4	0.3	0.8
	12	34.8	31.7	37.2	34.5	3.1	2.4	0.3
	13	24.8	27.6	29.0	22.5	2.8	4.2	2.3
	14	19.8	21.1	20.8	20.0	1.3	1.0	0.2
	15	25.4	24.4	27.9	25.5	1.0	2.5	0.1
S	16	10.7	11.2	11.1	9.7	0.5	0.4	1.0
	17	9.9	9.2	11.3	8.4	0.7	1.4	1.5
	18	12.1	11.7	12.3	11.4	0.4	0.2	0.7
	19	12.1	9.9	11.4	11.3	2.2	0.7	0.8
	20	12.7	11.1	10.9	13.0	1.6	1.8	0.3

L, M–L, M–S, and S represent grass carp of large, medium–large, medium–small, and small sizes, respectively. “Real” denotes the actual mass, “P” denotes the predicted value, and “M” denotes the mean absolute error. All values are expressed in g.

## Data Availability

The data can be accessed at https://github.com/pigcat88/FishKP-YOLOv11.git (accessed on 28 August 2025).
